# A Blockchain Secured Pharmaceutical Distribution System to Fight Counterfeiting

**DOI:** 10.3390/ijerph19074091

**Published:** 2022-03-30

**Authors:** Kavyan Zoughalian, Jims Marchang, Bogdan Ghita

**Affiliations:** 1 Computing Department, Sheffield Hallam University, Sheffield S1 1WB, UK; 2 Computing Department, and Advanced Wellbeing Research Centre, Sheffield Hallam University, Sheffield S1 1WB, UK; jims.marchang@shu.ac.uk; 3 Computing Department, Plymouth University, Plymouth PL4 8AA, UK; bogdan.ghita@plymouth.ac.uk

**Keywords:** blockchain technologies, pharmaceutical distribution system, zero-knowledge proof, Markov model

## Abstract

Counterfeiting drugs has been a global concern for years. Considering the lack of transparency within the current pharmaceutical distribution system, research has shown that blockchain technology is a promising solution for an improved supply chain system. This study aims to explore the current solution proposals for distribution systems using blockchain technology. Based on a literature review on currently proposed solutions, it is identified that the secrecy of the data within the system and nodes’ reputation in decision making has not been considered. The proposed prototype uses a zero-knowledge proof protocol to ensure the integrity of the distributed data. It uses the Markov model to track each node’s ‘reputation score’ based on their interactions to predict the reliability of the nodes in consensus decision making. Analysis of the prototype demonstrates a reliable method in decision making, which concludes with overall improvements in the system’s confidentiality, integrity, and availability. The result indicates that the decision protocol must be significantly considered in a reliable distribution system. It is recommended that the pharmaceutical distribution systems adopt a relevant protocol to design their blockchain solution. Continuous research is required further to increase performance and reliability within blockchain distribution systems.

## 1. Introduction

Counterfeiting in the pharmaceutical industry is a major global issue, which causes fatality for hundreds of thousands each year [1]. Counterfeit drugs can cause a further financial burden on the healthcare system as they sometimes worsen patients’ health. They have between 10% and 30%of the global market share in the medical industry due to the ineffectiveness of monitoring agencies [1,2,3,4]. Moreover, for the U.K., this could soon be escalated further by the impact of Brexit, as the transition means that the U.K. will no longer be under the protection of the Falsified Medicine Directive (FMD) [5]. U.K. officers identified over 3 million counterfeit medicines and medical devices valued at over £9 million, with only seven criminals arrested [6], showcasing the effectiveness of the current monitoring system. According to recent investigations, the current pandemic has also helped boost the problem we face. Interpol’s investigation in March 2020 suggests that the sale of medicines and medical products, particularly related to the COVID-19 pandemic, is on the rise, with over 2000 online links advertising false pandemic-related medical items [7] the following operation (Pangea), coordinated by Interpol from 18 to 25 May 2021, resulted in thousands of illegally operating websites being removed [6]. Authors of [8] suggest that the current centralized monitoring systems are managed by a particular set of people with the risk of a single point of failure, e.g., a recent incident of the outage of Facebook services due to unintentional faulty configuration of the centralized servers [9] is a reminder of possible failure point in any centralized systems. The centralized systems are mutable and require a vast majority of trust from the responsible parties for handling such systems to ensure the integrity and ingenuity of the stored data; consequently, it is justifiable to assume that a centralized system is usually an easier target for cyber threats than a decentralized system. Monitoring and enforcing regulations on fighting against counterfeit medicine is costly and time-consuming, particularly for developing countries [5,8,10]. In addition, the lack of transparency throughout the pharmaceutical distribution system, from the manufacturers to the wholesalers, retailers, hospitals, and pharmacies, has been the main drawback of the current solutions [3,4,8]. These factors contribute to the need for scalable, transparent transaction records of the medicines and, at the same time, a secure decentralized monitoring system for pharmaceutical distribution. In contrast, such a system reduces the trust factor between the participants. Hence, this paper tackles some issues related to a decentralized monitoring system. It proposes a solution for a trustless distribution monitoring system to fight against counterfeit drugs using a variation of the zero-knowledge proof protocol (ZKP).

The rest of the paper is organized as follows: Section 2 covers a literature review and analysis of the current research, Section 3 includes the methodology adopted for designing the prototype, followed by the design and implementation of the prototype in Section 4. Section 5 discusses the testing and findings of the designed prototype system. Section 6, Section 7 and Section 8 discuss the findings, critical evaluations, and provide conclusions, respectively.

## 2. Background and Literature Study

This section critically evaluates and analyses existing solutions and technologies used to fight against counterfeited drugs in the pharmaceutical industry, to build the readers’ understanding of the necessity of the current research; an investigation is performed to identify the scope of the methods, gaps, advantages, and disadvantages of some existing practices and solutions in the pharmaceutical world in the distribution sector. Further down this section, the paper investigates types of blockchain systems and different consensus protocols relevant to the solution proposal. The blockchain consists of several components; the network type of the system, the genesis node, participating nodes, the distributed ledger, and the consensus protocol of the system. The core architectural components of blockchain include:

- Nodes—a computer or an entity within the blockchain network;
- Transaction—an event to create a new block within a blockchain system. It contains relevant data that need to be stored, e.g., transaction of medicine to a particular node or entity within the blockchain ecosystem. Each transaction consists of a recipient and a sender detail, medicine detail, making the flow of goods transparent;
- Block—data structure used to keep a transaction or set of transactions distributed to all nodes;
- Chain—also known as the ledger, series of blocks in a set order;
- Miners—also known as full nodes that perform block verification process;
- Consensus—a set of rules for the verification process and blockchain operations.

**Figure 1:**
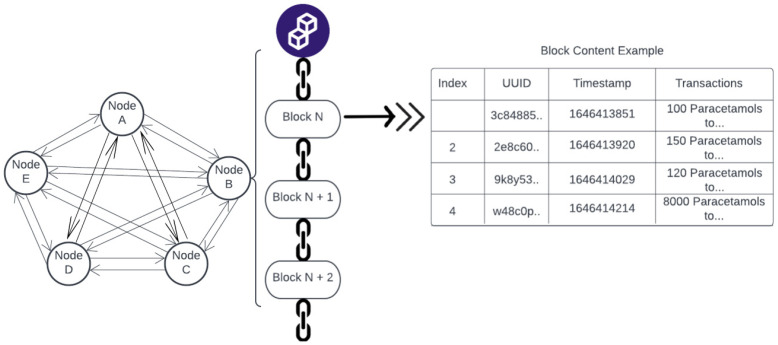
Example of blockchain components.

Blockchain is a decentralized distributed ledger-based technology model. It consists of a chain of blocks containing transaction information [11]. The chain becomes invalid if the link is broken [12]. A blockchain system is an immutable distributed database; once the information is stored, it cannot be tampered with. All the nodes in the network store transaction information as interlinked (append-only) chains, so it is transparent for the user if the chain ever breaks. Figure 1 illustrates an example of blockchain components. A block typically contains a timestamp, unique identifier ID (UUID), and other relevant information, including a list of transactions. This level of transparency provides a significant level of detail for monitoring purposes. The network is heavily dependent on the record-keeping of the nodes within its network. Since there is no centralized server or authority to ensure the integrity of the data, each node keeps an identical copy of the blockchain and comes to an agreement as a whole; each time, a block is added to the chain using a consensus protocol.

### 2.1. Existing Solutions

#### 2.1.1. RFID and Barcode Technology

Mass serialization using radio frequency identification (RFID) and barcode technology have been used widely for the past few decades in the healthcare industry to track medicines through the supply chain. RFID is more costly to implement than barcode technology, and it is much more effective against cloning and fake identification. This has helped reduction in medication and diagnosis errors in pharmaceuticals [13]. Barcode is a much older technology, and so is its efficiency in product tracking [3,14]. However, the work described by the authors of [3] elaborates on a cheaper manufacturing technique of RFID compared to the past, making RFID a better solution for delivering improved operational performance. The aforementioned technologies are necessary for the identification of packages and complementary for developing an effective monitoring system.

#### 2.1.2. Holographic Technology

Unique holographic packaging is another solution against counterfeit products. Although it is not impossible to clone such packaging, the manufacturers use holographic technology to ensure the legitimacy of these packages. However, due to reprinting and repackaging by retailers, this method can often be costly and inefficient [8]. Furthermore, it leaves room for errors and malicious intentions.

#### 2.1.3. The Falsified Medicine Directive (FMD)

The European Council adopts the Falsified Medicine Directive (FMD) to introduce measures to fight medicine falsifications and ensure medicine safety within Europe [15]. This is an example of a centralized monitoring system where the FMD servers control the monitoring for the flow of medicine in Europe. From the end of 2020, the United Kingdom is no longer under the protection of FMD; therefore, an alternative may become necessary for the United Kingdom.

Most or all the existing solutions do not provide a transparent picture across the supply chain. It leads to mistrust, data falsification, un-traceable, inefficient, opaque, and provides an opportunity to manipulate where and when possible. This idea is confirmed by the reports highlighted in the work of [16].

### 2.2. Blockchain Solutions

This section consists of the current solutions, research, and studies on blockchain and supply chains in various industries and their benefits.

In addition to cryptocurrencies and non-fungible tokens (NFT), researchers and government bodies notice other advantages to a decentralized ledger. U.S. Food and Drug Administration (FDA) published an announcement to develop an improved track and trace system for the supply chain of medicine. It is set to come into effect in 2023. The new proposed system includes technologies such as blockchain [17]; this shows that the benefits of using blockchain technology are recognized within the government bodies. The distributed ledger increases trust and results in economic efficiencies; established pharmaceutical companies such as Pfizer, Amgen, and Sanofi have been exploring using blockchain to document the testing of new drugs to speed up the creation of new medicines and their delivery to the market. Patientory and Coral Health are also considering allowing patients to control the data stored and track the medicine type and quantity they have received over time, taking advantage of the transparency this technology brings [18,19].

Additionally, other supply chains consider blockchain technology; as an example, Walmart participated in research to use the blockchain system, improving transparency and faster processing in their food supply chain [20]. The authors of [3] introduce the need for blockchain technology in Taiwanese pharmaceuticals by proposing ‘Gcoin’. This suggests that using distributed ledger can help implement a monitoring system that ensures the integrity of the drugs, improves the quality of the products, leading to better security and health of consumers; nevertheless, this paper limits the technicality of the consensus features the system could provide. Similarly, the authors of [21] propose a real-time remote monitoring private system based on the Ethereum protocol that allows physicians to record patients’ up-to-date health history. However, both articles lack the consideration of assuring a genuine supply of products’ history in the medicines’ distribution chain.

One of the implementation decisions to be made is the nature of the transaction environment and the consensus protocol they adopt. The authors of [4] suggest that private blockchains are more secure than the alternative. This way, legitimate participants are granted access to participate on the blockchain. The work of [8] proposes PharmaCrypt, an application tool using Ethereum private blockchain system. The application uses Amazon Web Services (AWS) and smart contracts, and in the model, products can be created and transferred between accounts. Mobile devices running the application can work as a barcode scanner to create new assets assigned with a unique identifier number; this promises implementation of the current technologies in use into an up-to-date supply chain system. The author indicated some scalability issues with their solution and further development requirement. It is argued that private blockchains are not genuinely decentralized as the participating nodes must be authorized to be a part of the network [22]. However, in a distributed system, the nodes are required to be authenticated by an authority (or, in this case, the genesis system); to ensure the integrity of the incoming nodes to the rest of the network.

#### 2.2.1. Private vs. Public

In 2009, Satoshi Nakamoto implemented the first blockchain as a public ledger for cryptocurrency using the proof of work (PoW) consensus technique [12]. Since then, blockchain technology has been evolving to create immutable chains meeting different criteria. Many blockchain solutions are currently under development to meet different needs that include providing transparency, integrity, traceability, and auditability. Blockchain systems usually fall into four categories: permission/private blockchains, permissionless/public blockchains, consortium blockchains, and hybrid blockchains. A private blockchain is an invite-only type of network. Each node on this type of network gets authorized by a group (or an individual/genesis system) before gaining access rights. There are several use cases of a private chain in supply chain management, global financial trade, retail, healthcare, and more. Permissionless chains are, however, open to join and leave on demand by the public; also referred to as public chains, it is ideal for cryptocurrencies and e-voting systems. A consortium chain is similar to a private blockchain, but the key difference is that it gets governed by a group of entities rather than one. It can be used in similar industries as a private blockchain would. A hybrid blockchain combines public and private features, so it is a blockchain accessible by the public, but a smaller group of authorized nodes do the modifications.

Public blockchain gives the pseudo-anonymity feature to keep the network’s identity unknown [23]. They usually consist of full nodes and simple nodes. Simple nodes can send and receive transactions; it is not necessarily required for a simple node to store the ledger or validate a transaction; in a proof of work (PoW)-based blockchain system, simple nodes are the users of the network. A full node must store the full blockchain, participate in the consensus and validate each block; full nodes in the same PoW network are the miners. A private blockchain network is an invite-only network, where the new nodes go through an authentication through the authority before joining the network. Transactions are usually visible to authorized users, whereas, in a public ledger, transactions are visible by everyone in the network; this increases the data privacy of the transactions in a private blockchain network [24]. Usually, each node in the network uses asymmetric encryption keys for digital signatures and authentication of their identity to the rest of the network. In a public ledger, these asymmetric keys are the primary identifiers of the node [25]. Permissionless or public blockchains are ideal for anyone to join to validate the blocks or create a new transaction; however, scalability is an issue factor due to its availability to the public [26].

Cryptocurrencies and NFT are the popular public blockchain as the public is permitted to participate in block creation and consensus making. Bitcoin and Ethereum are the most famous examples that fall in the same category; they both rely on public verification (miners) of the transactions [27]. Miners in a blockchain network consume much computational power; they require high electricity and hashing power, making them an expensive solution to implement and maintain. According to the work of [28], Bitcoin, which runs on a proof of work consensus, consumes 83.23 TWh of electricity, equivalent to the power consumption of Finland. According to the work of [29], Bitcoin solely uses more electricity than Argentina [28]. For a pharmaceutical distribution system, a private chain is ideal for authorizing and authenticating the nodes entering the network and holding them accountable for their integrity. If a validator attempts an attack on the network, meaning a validator should try to compromise the network with a new set of data or defies integrity, a penalty mechanism slashes the node’s stake and ejects the node from the network; Algorand is an excellent example of proof of stake (PoS) in a distributed network [30] introduces a pure proof of stake (PPoS) protocol and private blockchain, addressing security concerns of such system such as the ’long range attacks’ [31] with forward-secure signature. This protocol mechanism is an example of a ‘consensus’ protocol. The problem arises when the participants with the majority of the stake either become the target to intruders or dictate false information tampering with the integrity and purity of the data input.

#### 2.2.2. Consensus

From the literature investigated, a consideration of decision-making protocol to increase the network’s trust-based integrity and reliability was unidentified. This section includes some of the relevant consensus protocols adopted by different blockchain technology for block verification and validation process, canvassed, and compares their relevance to the pharmaceutical distribution supply chain.

In real-world, day-to-day interactions, people decide whom to interact with based on their reputation and how much they trust them; similarly, merchants in the market build reputation and trust-based on long-term fair trade between them [32]. In a distributed ledger, the problem arises when nodes in the network need to trust one another on the integrity of the data received. The authors of [33,34,35] demonstrate a zero-knowledge proof protocol (ZKP) where the nodes can agree on the knowledge of data D by proving K, where K is directly relative to D, without revealing the actual data. An application of ZKP is the circuit computations model. A random subset of the encoded version of the data requested from the verifier and the prover provides the subset of the requested information to be verified.

As part of the consensus of the blockchain system, all the participating nodes must agree on the appended block, so another problem that the system needs to address is when the system splits into multiple nodes with different responses in the verification process. The authors of [36,37,38] introduce trust-based consensus protocols with a reputation scoring system; these papers suggest a rating system for the nodes in the network based on their participation in the consensus. An alternate way to calculate a node’s trustworthiness is using the Markov model [39]. The authors of [40] discuss the use of proof of authority (PoA) protocol; the ‘authority’ becomes the final decision maker; however, it seems adrift from the idea of decentralization and also a risk of a single point of failure, since the authority is known to the participating nodes in the network.

An in-depth analysis of pre-existing literature has led to an overview of the blockchain technology and relevant features it can provide before conducting the programming element of the research work presented in this paper. There are suggestions to improve the management of the supply chain through the use of technology [19]. One way to tackle this issue is using blockchain. Using a decentralized ledger reduces operational inefficiencies and increases overall security. The blockchain network can be Byzantine fault-tolerant; this gives full transparency over transactions happening in real-time. Allows participants to monitor the data, which, in the end, concludes to patients receiving genuine products that route back to legitimate manufacturers [41]. The paper aims to adopt variations of the literature covered, such as the work of [35], the ZKP circuit computation model, and the use of the Markov model to make a consensus decision once the system splits into groups with various responses. The paper established the suitability of a private blockchain network environment for a pharmaceutical distribution system, so the nodes are required to go through an authentication process with authority (genesis system).

This paper investigates some of the current solutions to counterfeit drugs and recent issues and proposes a transparent distribution system by considering the reputation of the nodes and by contemplating their security measures and their needs.

## 3. Design and Setup

### 3.1. Current Pharmaceutical Supply Chain

**Figure 2:**
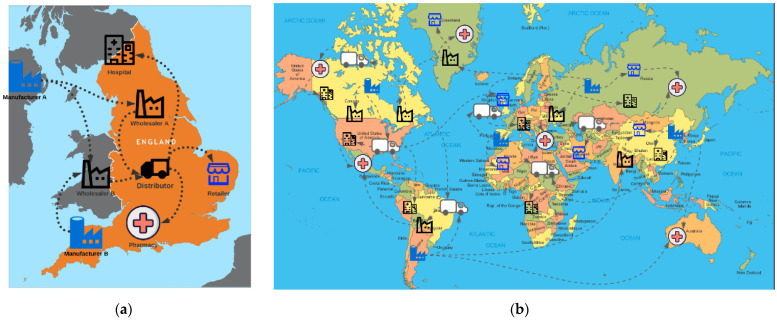
Pharmaceutical distribution system examples. (**a**) Distribution of medicine in the U.K.; (**b**) distribution of medicine worldwide.

The current supply chain involves the flow of medicine transfers from manufacturers to wholesalers, down to the consumers. Typically, the wholesalers place their order with the manufacturers; they receive the order and deliver the products. Wholesalers then receive the products and directly distribute them to pharmacies, hospitals, and retailers. They often provide them to the distribution centers, where they handle delivery of the products to providers. The entities then provide patients with the medicine. This is illustrated in Figure 2a,b. The manufacturers and wholesalers do not usually offer the medication to the consumers directly, and neither do the distributors. The distribution system of the drugs may vary from Figure 2a,b; however, this is a typical example of the current medicine flow in the supply chain.

Each entity in this supply chain has its supply chain system, or a vendor managed inventory; generally, there is minimal shared data between these systems for security or policy reasons, resulting in a lack of transparency and trust; due to the reliability of the system, what if the data are compromised at a later stage on the centralized server? Technical issues resulting in data loss? It is important to ensure the integrity of the quantity and type of medicine held by each entity that can prevent external counterfeit medicine from being issued to third parties or consumers. The design of the prototype in this paper attempts to address these issues. The implementation of technologies such as RFID, barcode scanners, and holographic packaging can complement this system at a further stage.

The paper proposes a proof of concept; for an industry-level implementation of the proposal, regulations surrounding the pharmaceutical supply chain must be considered, such as ICH Q10, Q9, and ISO 9001.

### 3.2. Proposed Testbed

The network architecture design of the prototype is to simulate a peer-to-peer network; the nodes in the network are directly connected as a core requirement of a blockchain network, each node representing participants in a distribution ecosystem. Participants such as hospitals, manufacturers, and government bodies can be part of the consensus decision-making process by being involved in the peer-to-peer network.

### 3.3. System Configuration

**Figure 3:**
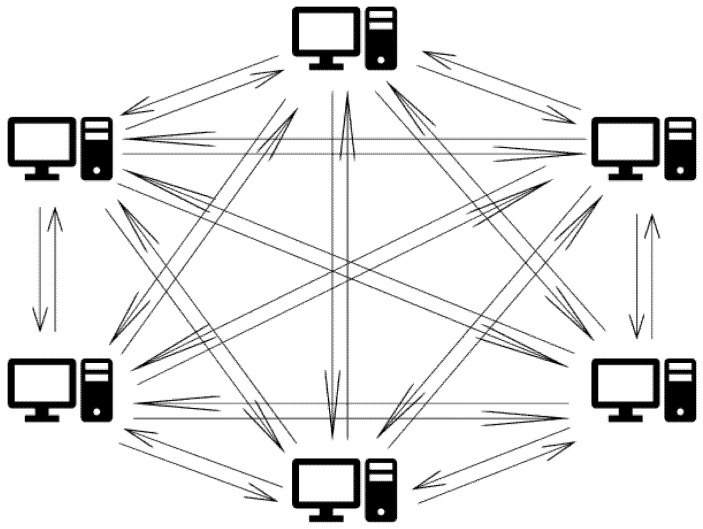
Network simulation infrastructure.

One router, two windows machines, and three Raspberry Pi were used for a supply chain network simulation. The limitations of the recent pandemic restrict the ability to implement the prototype on a larger scale. The network diagram of this simulation is shown in Figure 3. The specification details of the machines used in the setup, followed by the libraries and tools needed, are found in the following sections. The IP of the nodes is set to static and fixated to identify the nodes by their IP. The systems used during the testing are provided with detailed main configurations in Table 1.

### 3.4. Requirements

The first phase of the prototype model is requirement analysis, a set of functionality expectations that are expected from the solution.

#### 3.4.1. Functional Requirements

Overall functional requirements of the prototype are stated in the paper’s objectives. The requirements are grouped into two sections that include high and low-priority features to implement. High-priority functions are essential core requirements that we expect from the prototype, and low-priority functions are desirable functionalities that do not necessarily affect the core functionality of the aim of this paper. Time constraint has a direct impact on these requirements. The paper aims to meet the higher priority requirements first, as highlighted below.

*I. High-Priority Requirements:*

(a) New nodes should connect to the genesis system to join a peer-to-peer network;
(b) The application should implement a simple consensus based on a variation of the zero-knowledge model with reputation-keeping features;
(c) Use of Markov model for reputation calculation of the nodes with each round update of the reputation score;
(d) Secure communication of the nodes in the network with RSA encryption;
(e) The application should save and update the blockchain in real-time.

*II. Low-Priority Requirements:*

(a) Implement error detection within the block content by using Merkle root;
(b) Implement self-healing factors within the system by using the linear Merkle function;
(c) Transfer a list of nodes, blockchain file, and public key of the nodes to the new nodes;
(d) New nodes send their public key to the nodes in the blockchain network.

#### 3.4.2. Non-Functional Requirements

The non-functional requirements of the prototype are a group of conditions that complete the application. Similar to the functional requirements, this paper prioritizes the needs based on high-priority conditions. The application standardized a working prototype, and then, tried to include the low-priority conditions that are desirable for the application.

*I. High-Priority Requirements:*

(a) The application is expected to run on multiple Unix-like operating systems such as Raspberry Pi, which is a Debian-based operating system;
(b) The application to run on Windows machines;
(c) The application should address and prevent common security concerns.

*II. Low-Priority Requirements:*

(a) The application should minimize the use of ports for security reasons;
(b) The application should consider minimizing network traffic to improve the performance and efficiency of the prototype;
(c) The application should be compatible to run on any integrated development environment (IDE) and command-line user interface.

### 3.5. Python Programming Language and Other Tools Used

**Figure 4:**
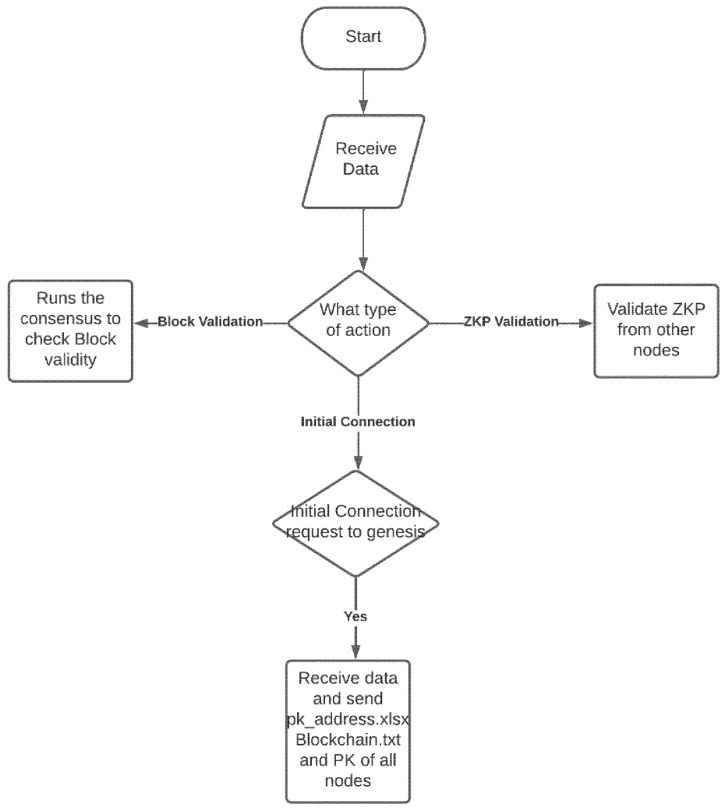
Receiving data.

The proposed solution is designed based on the Python programming language. Python was selected for its quick prototyping capabilities, open-source materials, and availability of various libraries, including external libraries such as socket, pandas, openpyxl, and xlrd. These libraries are used for communication between the nodes and data analysis. Using the threading module, the code divides into multiple threads to run simultaneously, allowing nodes to create a new block, receive new connections to the network and receive newly created blocks for validation, simultaneously as per the availability of the network and system resources. Additionally, this feature allows the application to interact with the users to create a new block and participate in the consensus at the same time. The aforementioned is illustrated in Figure 4.

The network architecture is peer-to-peer to maintain the values of the blockchain system; this ensures the prevention of a single point of failure within the network. The program is set to run on Spyder IDE, Thonny IDE, and command-line CLI. The following libraries are required to be installed:

1. Openpyxl;
2. Pandas;
3. Libatlas-base-dev;
4. Pycryptodome;
5. Xlrd version 1.2.0.

All libraries mentioned above are accessible and are installed using pip command. Libatlas is the only library that needs to be installed by apt-get command.

### 3.6. Encryption

PyCryptodome and hashlib libraries are used for the implementation of RSA encryption for the communication between nodes. The nodes in the prototype use RSA asymmetric encryption with a 2048-bit key with 0AEP padding. NIST SP 800-57 rev 5 indicates the use of this key-size with 112 bits of security strength is “Acceptable”, which is considered secure by NIST standards for the proof of concept of this prototype. The keys are generated using a separate piece of code to the main program, and it is stored on the local storage system of the node with a.PEM extension assuming that each node has its private key and public key, using RSA encryption 2048 key length before joining the network. All the communication between individual nodes is encrypted using the mentioned encryption method. All the encryption keys are stored in the program’s current directory in the ‘RSA’ folder.

### 3.7. Nodes

This section will consider the differences in operation between the genesis node and the regular node. This section illustrates the functionality of the nodes and their activities.

**Figure 5:**
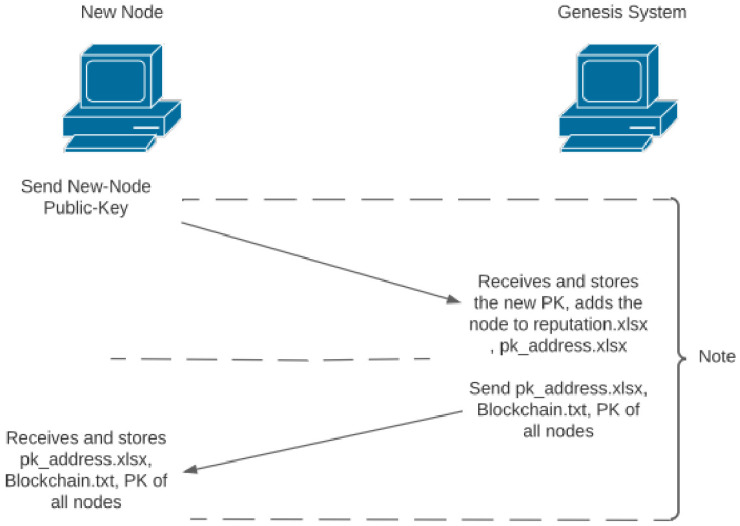
Initial connection and key exchange.

Executing the designed blockchain application initiates five processes to run simultaneously. These processes, apart from the main_menu function that interacts with the user, includes the recv_PK() function, which is the initial request to the genesis system illustrated in Figure 5, recv_new_PK() function to receive the new public key of the new node entering the network, recv_new_block() function to receive new blocks created by the nodes in the network, zero_knowledge_receive() to participate in the consensus decision-making process and rcv_zkp() function that receives other nodes responses for the consensus. The program first asks for the node’s IP address and checks to see if they have the node’s private key in the designated directory; if so, the user is presented with the main menu. Its IP address identifies each node; each node can represent an entity in the pharmaceutical supply chain network.

**Figure 6:**
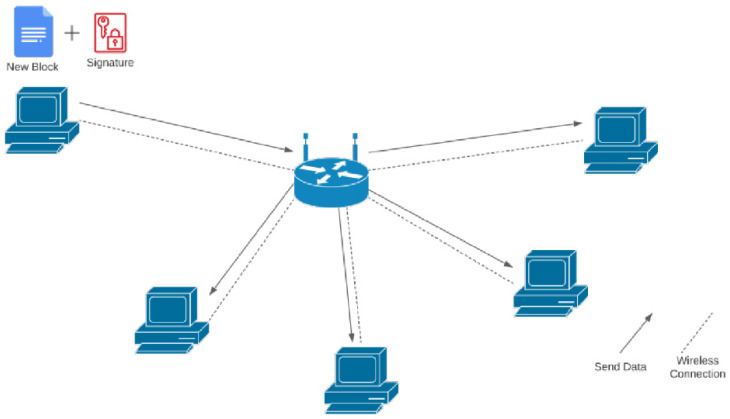
Block creation and digital signature.

An initial node in the system, also known as the genesis node, is the sole node running on the network at the beginning. The node can produce blocks, and as there are no other nodes in the network, the consensus returns null false, which results in storing the block recorded in the Blockchain.txt file. The new node that wants to join the network is assumed to have the public key of the genesis node; they can initiate the request on the main_menu function called ‘Initial connect’, the program then asks for the IP address of the genesis system to connect. The complete connection initiation is demonstrated in Figure 6, which illustrates the steps taken by the new node and the genesis node to complete acceptance of the new node to the network. Once the new node joins the network, it goes through the pk_address.xlsx file to check the nodes that are in the network; the new node sends its public key to all the nodes in the network, other nodes in the network receive the new public key as a thread using recv_new_PK function, adds the public key to the designated folder and enters the relevant data to the pk_address.xlsx and reputation.xlsx. All the incoming nodes are expected to go through the genesis system to join the network; otherwise, the regular node and the genesis node operate the same way.

### 3.8. Initial Connection

The new node only needs the genesis systems’ public key to initiate a connection to the network. The initial contact begins with the new node sending its public key to the genesis system; the genesis system then stores the new node public key in the designated file, adds the new node to pk_address.xlsx and reputation.xlsx; the next step would be for the genesis system to send a copy of the pk_address.xlsx, Blockchain.txt and public key of all the nodes in the network to the new node. The new node receives and stores the files accordingly. The purpose of these files are mentioned below:

1. Pk_address.xlsx—keeps the IP and public-key path for all the nodes in the network. The public-key directory path helps for faster access to the file;
2. Reputation.xlsx—reputation file contains reputation score and responses for each node (described in Section 4);
3. Blockchain.txt—is the distributed ledger that stores all the blocks created in the network.

### 3.9. Block Creation

A block contains all the relevant information the pharmaceutical distribution system needs. The block content includes the index number of the block; this depends on the number of blocks currently produced in the Blockchain.txt file. A universally unique identifier (UUID version 4) randomly generated using the UUID library to identify the block uses uuid4 means there are no relations between the block content and the unique ID; this prevents an intruder from gaining information about the block content. The UUID version 4 has possibilities, of which 2 satisfy our need for the 128 prototype system. The block contains a timestamp of the exact time the block was created and a list of transactions for multiple entries if the node decides to make multiple transactions in a block. Additionally, the block contains the medicine type, date of manufacture, expiration date, the quantity of the medicine involved, receiver, and the previous hash. For the Merkle tree implementation, the block would also contain the Merkle root of the block content and the Merkle root of the blocks created previously.

Any node in the network can create a block; this initiates with a node that wants to make a transaction of drugs to another node in the network; for example, a manufacturer who wants to send drugs to a wholesaler must create a block in the system. The entity starts with entering the necessary information for the block content, and the node then submits the block in JSON format along with its digital signature for approval by other nodes in the network; the rest of the nodes come to a consensus decision to either accept or reject the new block. If the block is affirmed, it is appended to the Blockchain.txt file in the designated directory.

Some of the block creation process challenges are the assurance of the integrity of the block content, the honesty of the nodes in the network, and the correctness of decision-making in the consensus process of the system.

### 3.10. Digital Signature

**Figure 7:**
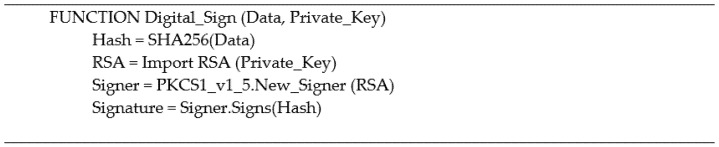
Digital signature pseudo code (sign).

**Figure 8:**
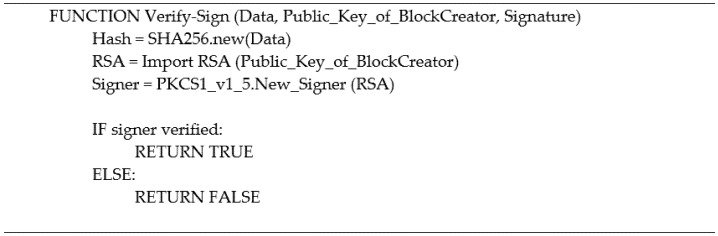
Digital signature pseudo code (verify).

Digital signatures are essentially the fingerprint of the data creator to verify the authenticity to the recipients that the data are not altered, and the genuine entity is performing the block creation. The signature is produced by the block creator, using its own RSA private key to encrypt the block content; this process is performed by the Digital_Sign function as shown in Figure 7, providing the data to be encrypted and the private key of the block creator. There are two steps to this process; firstly, the block creator sends the original data; in this case, it is the block content and the digital signature to all the nodes in the network; this is illustrated in Figure 6. The nodes in the network are a list of IPs stored in the pk_address.xlsx. The second step of the process is where the nodes in the network receive the original data and the signature; the nodes use the Digital_Verify function demonstrated in Figure 8, to perform the digital signature verification process providing the data obtained, the public key of the block creator, and the signature received.

Using digital signatures is a method to ensure the authenticity and integrity of the block content. The block creator can only perform the digital signature since they are the only node that hold the corresponding private key. The nodes must keep their private keys secure to prevent exposure to unauthorized users impersonating their identities.

### 3.11. Further Improvements

For better fault detection and usability, this paper suggests some features to be implemented on the blockchain distribution system; this includes the use of ‘Merkle Tree’ and ‘Application Programming Interface (API)’ as described. The implementation of these improvements are not necessary for the operation of the prototype system and may not be implemented due to time concerns.

#### 3.11.1. Merkle Tree

In a supply chain for a pharmaceutical distribution system, it is expected that there are a vast number of transactions that need to be stored in the blockchain system. Initially, the system uses the previous hash stored in the content of the block to check the data entry is not modified; suppose an entity in the network wants to examine the information integrity within its ledger and finds a modification, the entity then needs to request the whole file from another node to compare against and replace the modified content. Furthermore, it is possible only to examine the hash of the file to make this process easier; however, how does the node identify the altercation? It will still require a request to another node to receive the entire Blockchain.txt file. This is an inefficient method and causes unnecessary use of the network bandwidth to receive this information every time there is an altercation in the system. An alternative solution is the use of the Merkle tree.

A Merkle tree is a data structure of hashes, also known as binary trees, used in a distributed system for efficient data verification. The purpose of the Merkle tree is to verify the content of a set of data by pairing the encoded hash of each content recursively until reaching the Merkle root. Merkle root is the mathematical method to verify the data on a Merkle tree to prevent altercation throughout the blockchain system. This method helps the system identify the exact content altered to request only the modified content from the most reputable node in the network.

This paper suggests two separate Merkle systems to identify the particular section of the blockchain that was modified. However, due to time constraints, it was impracticable to implement this feature in the prototype system.

- Merkle Hash of Blocks

**Figure 9:**
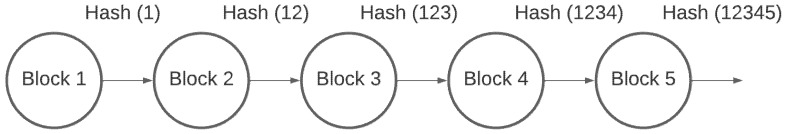
Merkle tree of blocks in the blockchain.

The first system is a linear function to store the hash of the block as a leaf 𝑛 + 1 where the Merkle root of this function is the pair of 𝑛 and 𝑛 + 1, and the Merkle root becomes the new 𝑛. This is illustrated in Figure 9, the hash of Block 1 and the hash of Block 2 are hashed to become Hash (12), which can be stored in Block 3, this would be the new Merkle root and can be represented as 𝑛, then this is encoded with the hash of Block 3 to become Hash (123) and so on. This method is used only to compare the Merkle root with other nodes to identify the block modified. If the hash is different from the one stored in the block, then it has been changed.

- Merkle Tree of Block Content

**Figure 10:**
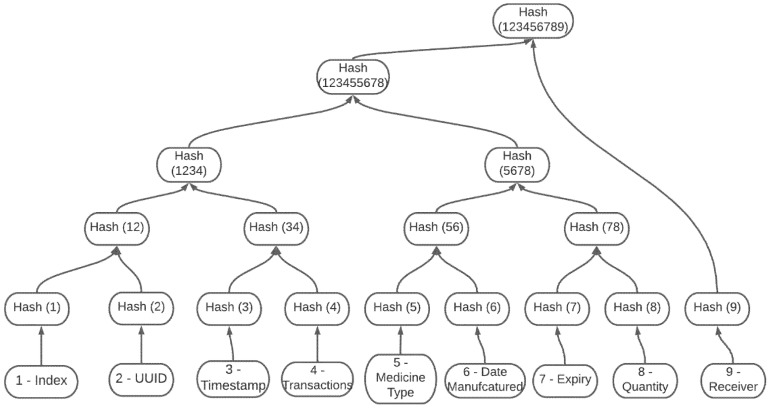
Merkle tree of block content.

The second system is the Merkle tree of block content illustrated in Figure 10; this system structures a Merkle tree for the block’s information to identify the exact content within the modified block. Each leaf of the tree represents every data stored in the block; the system can make its way from the top of the tree to identify the altered content.

#### 3.11.2. Application Programming Interface

APIs can be built on this system by developers to interact with the blockchain distribution system and pull-out information based on specific requests from government bodies and supply chain entities. APIs can help non-technical users to be able to interact with the blockchain system to pick out necessary information they may need. For example, the government body may need to know how much medicine is produced at a manufacturing level to monitor the outpost of the medication to the wholesalers. If the manufacturer outposts more drugs than it makes, then precise counterfeiting is identified.

Thus, the paper focuses on designing a prototype for simulating a pharmaceutical distribution blockchain system. Moreover, the study developed additional features to be included, improving fault tolerance and usability of the system. To complement this section, in the next section, the paper introduces an algorithm for the network to make a consensus decision on storing the next block in the system.

## 4. Zero-Knowledge Proof and Markov Model

Using zero-knowledge proof and keeping the network private are the additional measures put in place to ensure the security and partition tolerance of the system, which are briefly mentioned in the synopsis of the literature review.

PoS and PoW are the most popular consensus mechanisms; however, other methods are available to reach a consensus decision. In this section, the paper will discuss the proposed consensus mechanism of zero-knowledge proof and the use of the Markov model in the prototype system.

### 4.1. The Basic Idea of the Consensus Decision Making

Assuming the current pharmaceutical supply chain system becomes a distributed ledger, what is the permission protocol for creating a block? Does everyone have permission to write a block onto the system? If so, how does the system ensure the integrity and consistency of the data input?

A blockchain system is a method to implement a state machine replication; at any given time, the system stores a state of the system. As a result, the user can access an updated state of the system in real-time. Certain requirements must be met to achieve these criterium. In theoretical computer science, the CAP theorem, also known as Brewer’s theorem [42], states that a distributed system can only deliver two out of three characteristics. CAP theorem characteristics are; consistency, availability, and partition tolerance. Consistency implies that the exact data are accessible by all nodes at all times. Availability means that the network can resume operation at all times, meaning the system is always available and accessible for use without any malfunctions. Partition tolerance in a distributed system is where the network operates accurately even with conflicted responses by the nodes.

Typically, there are two types of failures a node may experience. The first is where a faulty node has crashed or, for whatever reason, becomes unavailable. The second type is where the node is compromised and exhibits inconsistent behavior arbitrarily. Consensus is an algorithm that reaches a distributed agreement between distrusting nodes for the final state of the system. A compromised node can affect the consensus decision of the network; therefore, the system must tolerate this behavior and ensure partition tolerance through the consensus protocol. Since the nodes in the network do not fully trust each other, a zero-knowledge approach is implemented.

### 4.2. Zero-Knowledge Proof

Zero-knowledge consensus is a Byzantine fault tolerance-based protocol that allows a network to reach an agreement on the integrity of the data received without revealing the actual data in any way.

This paper’s variation of the ZKP protocol involves the encoded hash of the new block that needs to be verified. In a pharmaceutical distribution system, the integrity of the distributed data and the partition tolerance of the network is crucial; in other terms, the nodes in the network must check the exact same data are distributed in the network and what gets stored in the blockchain is the same throughout the distribution system. The hash of the data is the exact same throughout, and a slight modification of the data can change the whole hash function; therefore, if the hash of the data is the same in all the nodes within the network, it indicates that the integrity of the distributed data is met.

However, sharing the hash of the data with verifiers is not exactly a zero-knowledge consensus; with the constant improvement of processing power, the hash function of data may become vulnerable to pre-image, brute-force, and collision attacks. In addition, sharing of the whole hash function by each node increases network traffic and processing of the verification of the system. The variation in the prototype imposes the use of a single positional character of the SHA256 hash function, meaning that proving a positional character of the hash increases the possibility of the integrity of the hash function of the incoming data. SHA256 hash includes 32 bytes and 64 in hexadecimal character length, which means each byte in SHA256 could have 25,632 possible values. Therefore, the prover has a 1/25,632 chance of guessing the correct character requested as a challenge by the verifier unless they have received the correct data. For security purposes, the verification process occurs 10 rounds before the verifier decides to accept or reject that the data received by the prover is accurate, which means the chances to be verified becomes (1/25,632)10.

#### 4.2.1. Honest Verifier

**Figure 11:**
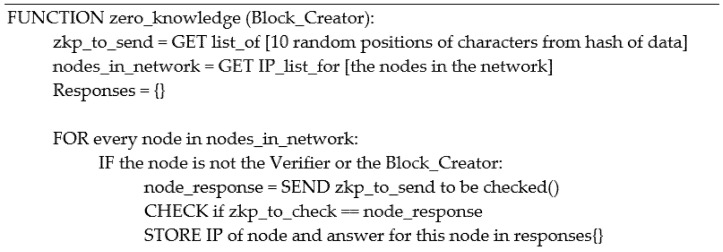
Pseudo code of verifier for zero-knowledge challenge.

**Figure 12:**
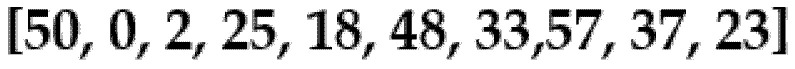
Example of positional characters of the hash value to be verified.

The honest verifier demonstrated in Figure 11,sends a list of 10 random positional numbers to the prover as shown by the example in Figure 12. The prover then sends back the completed task as a dictionary with the characters as the value and the number of each position as the keys. The honest verifier then checks the accuracy of the challenge and either returns true or false.

#### 4.2.2. Honest Prover

**Figure 13:**
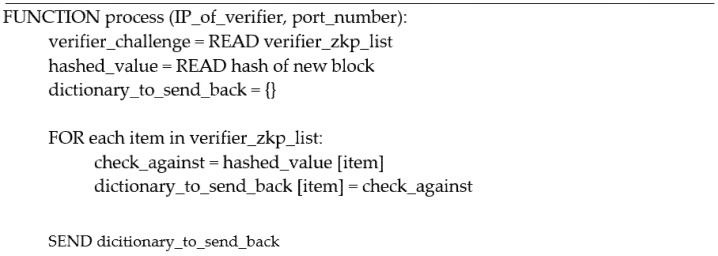
Pseudo code of prover for the zero-knowledge challenge.

**Figure 14:**
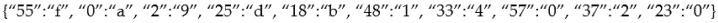
Example of the completed positional hash value of ZKP.

The honest prover, as shown in Figure 13 receives a list of 10 numbers, then processes the list in a for-loop adding the character and its position to an empty dictionary in JSON format. Once the for-loop is completed, the prover has completed the challenge and sends back the dictionary to be verified, as demonstrated in Figure 14. The JSON format enables the system to store and send the dictionary more efficiently; otherwise, the format is changed to a string by default.

This is single-round verification by all the nodes in the network to reduce network traffic and complexity in the consensus procedure. The protocol waits for all the nodes in the system to respond before making a final decision to send back to the block creator. There are two possible outcomes for this voting system, the first is for all the nodes in the network to verify the integrity of the new block, where in this case, the voting system returns null false, and the final decision is sent to the creator is ‘True’, and that is the end of the consensus protocol for the current round. The second outcome is where the network splits into two groups; a group of nodes that return ‘True’ in the verification process and a group that returns ‘False’, which means that the verification process was incorrect. This is where the problem arises; the nodes must agree on the decision based on the reputation of the other nodes, a reputation score gathered from the previous interactions. As a result, the nodes in the network continue with the second part of the consensus and reach a decision based on the answer given by the trusted nodes from the reputation.xlsx file. The final decisions are then sent to the block creator, where the same voting algorithm occurs; if the voting system returns null ‘False’, the block is stored, and the answers are stored into the reputation.xlsx file if the network is split in the decision, then the second part of the consensus protocol is activated, and the final outcome is based on the answer of the most trusted nodes of the network.

### 4.3. Effectiveness of Zero-Knowledge Proof

The use of this zero-knowledge proof variation processes one round interaction between the prover and the verifier to distinguish between a node with genuine data and an imposter with minimal or no shared knowledge of the data. The number of character checks between the two nodes can be reduced or increased; however, 10 randomly chosen characters are justifiable for the prototype distribution system at this level. Pseudo randomness of the characters in the verification process makes each consensus round to be unique for each node in the network; therefore, possession of the original data is a necessity for a node to be verified. The consensus protocol waits for the voting procedure to be completed by all nodes before sending the final decision to the block creator. The block creator observes the response by all nodes; if all the nodes return null ‘False’, the block is stored in the Blockchain.txt, and the consensus is successful. If the network splits in the decision, the node proceeds to the second part of the consensus.

### 4.4. Importance of Reputation

The split decision can occur on two occasions; either the prover sending the odd response received different data from the block creator, or the prover is compromised and is acting maliciously. Once the nodes in the system split in the decision, it creates a dilemma in the network. This makes it vulnerable to 51% of attacks. To prevent this vulnerability, the consensus protocol only considers the response from the most trusted nodes in the network. The most trusted nodes are proportionate to the number of nodes in the network; to avoid chances of a single authority becoming the decisive node and to dictate the network, if the total number of nodes in the network is less than 4, the number of trusted nodes is 2; however, if the number of nodes exceeds or is equal to 4, 30% of the network is classed as the ‘trusted nodes’. The trusted nodes are rounded up and are based on the nodes with the highest reputation in the system. This paper adopts the Markov model for the reputation calculation of the nodes based on the integrity of the previous interactions between each node.

### 4.5. Markov Chain

Markov model is a stochastic model used on an active system with often behavioral changes to predict the probability of future states and events. The Markov chain is a Markov model used by autonomous systems where the state is entirely observable. The possibilities are calculated by observing the transition states; these are states based on the prior occurrences of an event illustrated in a transition matrix. The transition matrix can be used to score the reputation of the nodes in the system based on the history of interactions.

The prototype system records the answer for nodes at the end of each round and updates the reputation score accordingly. The states and reputation score for each node is stored in the reputation.xlsx file in the current directory, this file is not shared with any other node, and each node has its own copy of the file; this secrecy of the reputation file is inspired by the authors of [43], the study suggests that unpunished misdeeds evoke a need for justice restoration.

#### Reputation Score Calculation for Consensus:

In a consensus round, only the reputation score is considered and is compared with the rest of the nodes to identify the trusted group within the distributed blockchain network. The reputation score is calculated based on the assigned trust probability scores as shown in (1), and the matrix scores are provided in (2). The previous state and current state of a node is represented by $P_{s}\;\mathrm{and}\;C_{s},$ respectively. Moreover, when a node x is trustable, then T is assigned; otherwise, F is assigned as shown in (3). The probability score of 1, ½, ½, and 0 are given when the previous state $\left(P_{s}\right)\;$ is changed to the current state $\left(C_{s}\right)$ from T→T, T→F, F→T, and F→F. It means that when the state of a node is always yielding to the overall consensus outcome of the network, it remains highly trustable (T→T), and when the state of the node toggles (T→F or F→T) then the reliability of the outcome is reduced while the trust outcome is considered nil when the previous state and the current state remains not reliable, i.e., F→F. In this proposed trust calculation framework, the last six states of a node is considered for the consensus decision-making process during the block validation process. The recent history of the node about trust value is considered rather than considering the trust value of the entire history of the node’s state to provide a higher scope of joining during the participation in the consensus process. The overall trust score ($Tr_{score}$) at a given instant of a node’s state is calculated as shown in (4). Once the consensus protocol is complete, and the final response is sent to the block creator, the last step is to calculate the reputation score based on the outcome decision of the consensus protocol. The responses are stored in reputation.xlsx, and the reputation calculations are demonstrated below.

(1) Probabilityscore={PsCs is TT, 1PsCs is TF, 1/2PsCs is FT, 1/2PsCs is FF, 0

(2) $$P=\left[\begin{array}{cc} 00 & 01\\ 10 & 11 \end{array}\right]=\left[\begin{array}{cc} FF & FT\\ TF & TT \end{array}\right]=\left[\begin{array}{cc} 0 & 1/2\\ 1/2 & 1 \end{array}\right]$$

(3) $$f\left(x\right)=\{\begin{array}{l} T,\;Node\;x\;is\;Trustable\\ F,\;Node\;x\;is\;NOT-Trustable \end{array}$$

(4) Trscore=∑i=0i<nPsCsin, where n=6

**Figure 15:**
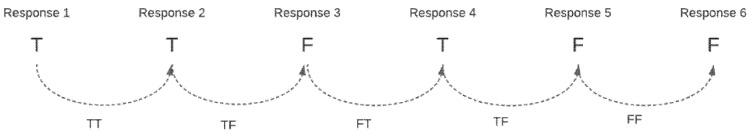
Transition states example.

Figure 15 represents an example of the transition states of a node. The transition probability matrix of a Markov chain, also known as the stochastic matrix, is calculated by (1) and (4), and the transition matrix of the example provided in Figure 15 is given in (2).

Since 𝑇𝑇 appears once and 𝑇𝐹 appears twice in Figure 15, so the probability of its occurrence is 1/6 and 2/6, respectively; similarly, 𝐹𝑇 appears once, and 𝐹𝐹 appears once, so the probability of occurrence is 1/6. Thus, the relevant probability scores that the system accounts for depending on the last six states of a node. The reputation score is a prediction of the honesty of the node in the series of last recent responses.

What is the reputation score of a new node? The system can implement one of the two approaches as a policy; either the new node joins the network with a zero-trust policy or provide the new node the benefit of the doubt and start with a 1/2 reputation score since there are no previous interactions with the new node the next response from this node can either be in the state of true or false. It appears to be acceptable to provide a 1/2 reputation score to a new node joining the network if the reliability and trust ability is not fully formed. However, it becomes possible for a compromised node to leave the network and re-join using different credentials to restore their reputation score, so it is dependent on the toxicity of the network environment to implement this method. Since the system is expected to run on a private network blockchain system, the paper suggests the second method to keep the nodes engaged with the consensus protocol.

**Figure 16:**
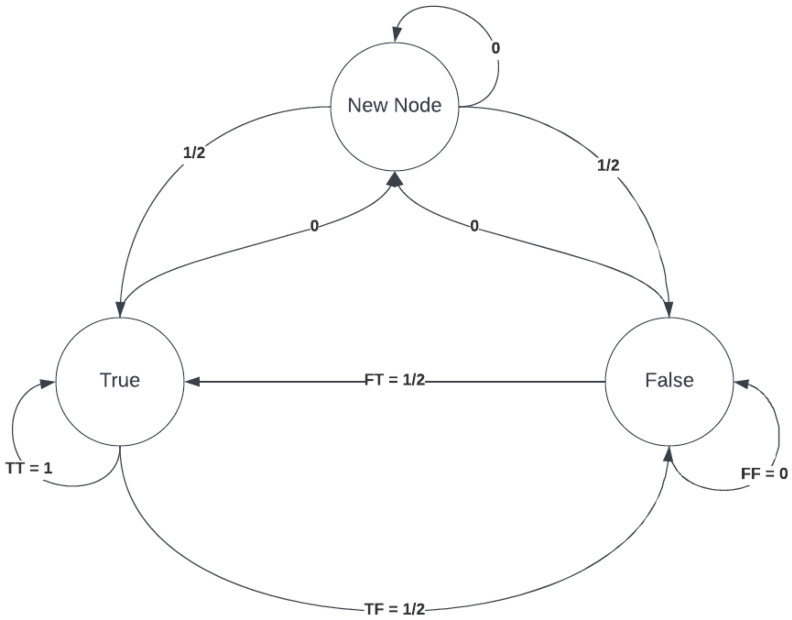
Markov model example diagram.

**Figure 17:**
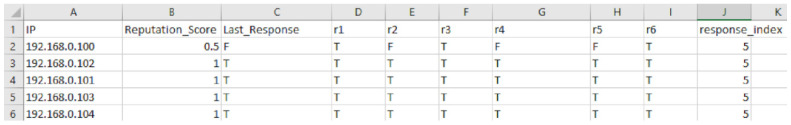
Reputation.xlsx file information format and score keeping of the nodes.

Figure 16 represents an example of the Markov chain.. The node’s last response has a direct impact on the reputation score; as the system is only concerned with the possibility of the node’s next response being true, meaning that if the last response is false, then the reputation score will be the probability of the transition 𝐹𝑇 or if the last response is true, then the score is the transition probability of 𝑇𝑇. The record-keeping and storage of the reputation are shown in Figure 17.

**Figure 18:**
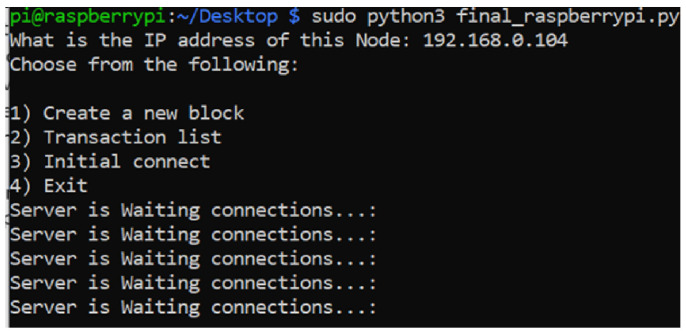
Starting the application.

The system collects and records six responses from the interactions to be able to produce five transition states, as portrayed in Figure 12. An honest node can suddenly be compromised by an intruder to commence malicious behavior; therefore, the most current five states of a node deem justifiable to estimate a reasonably accurate probability of the next true answer and keeps the reputation score up to date; however, this shows that the reputation score is only an estimate and prediction and there will always be an anomaly to this result. Figure 18 conveys the format in which the data are stored in the reputation.xlsx file. The system uses column J (response_index) to keep track of the position the last response was stored.

## 5. Results, Testing, and Findings

In Section 3 of the paper, a list of requirements was stated to create a smart pharmaceutical distribution system using blockchain technology. These include nine functional requirements and six non-functional requirements. This section analyses the implemented system to determine whether the conditions have been met.

### 5.1. Initial Connection

Once the prototype application initiates, the user is asked to input the node’s IP address where the system reads the RSA private key from the designated directory. This would be an initial authentication of the node. After the user is prompted with the menu, illustrated in Figure 18, the multiple threads start running simultaneously, as described in Section 3.7. The user is able to create a new block, see a list of transactions in the blockchain, initiate a connection to the genesis system, or exit the application.

Section 3.8 describes the initial connection procedure to the genesis system. The new node starts with the public key of the genesis node, initiates the initial connection request to the node, and submits its own public key. The genesis node stores the new public key with the file-name format of public_key-[IP address of the new node].PEM and starts keeping the reputation score of the new node, based on the later interactions with the node stored in the reputation.xlsx file. The genesis node then sends a copy of the Blockchain.txt file following with the pk_address.xlsx, which contains the IP of all the nodes in the network. The genesis node starts a for-loop sending the public keys of the nodes to the new node according to the order of the pk_address file.

Once all the files have been received from the genesis node, the new node creates a new entry for the nodes in its own reputation file and sends its own public key to all the nodes in the network.

### 5.2. Consensus

The application implements a smart consensus based on a variation of the zero-knowledge proof demonstrated in Section 4.3. The reputation keeping of the system and the decision-making of the network accordingly make this a smart system.

**Figure 19:**
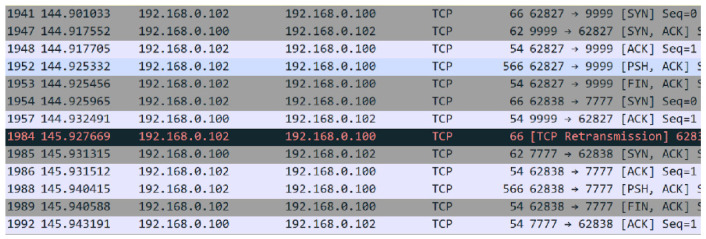
Initial block creation process transfer of block and signature from 192.168.0.102 to 192.168.0.100.

Figure 19 is a snapshot of the network traffic captured by Wireshark, exhibiting the first step of the block creation process; previously mentioned in Section 3.10, node 192.168.0.102 created and sent the block and the digital signature on separate ports for verification to node 192.168.0.100. Figure 19 demonstrates a TCP three-way-handshake for a complete transfer of the block data and block digital signature. The block is sent to port 9999 and the digital signature to port 7777, where the nodes accept and receive data as an active thread.

**Figure 20:**
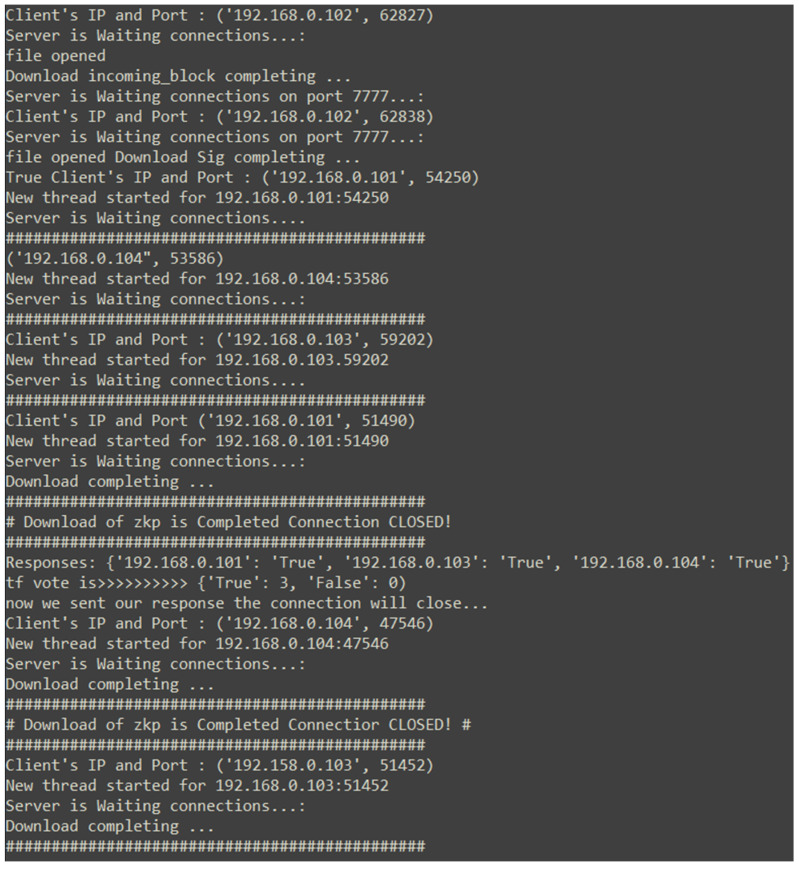
The regular node (192.168.0.100) receives the new block and the decision-making process.

**Figure 21:**
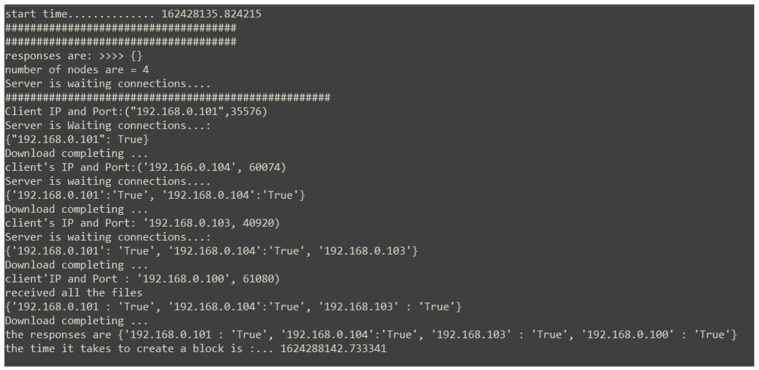
Responses and voting system on block creator’s side.

Similar to the process demonstrated in Figure 20, Figure 21 illustrates the same voting mechanism. The system shows no disagreements between the nodes; therefore, the second step of the consensus mechanism is skipped, the block is appended to the Blockchain.txt, the nodes’ responses and the new reputation score of each node are calculated, and stored in reputation.xlsx.

If the nodes disagree with each other, then the second phase of the consensus process activates, as demonstrated in Section 4.5.

**Figure 22:**
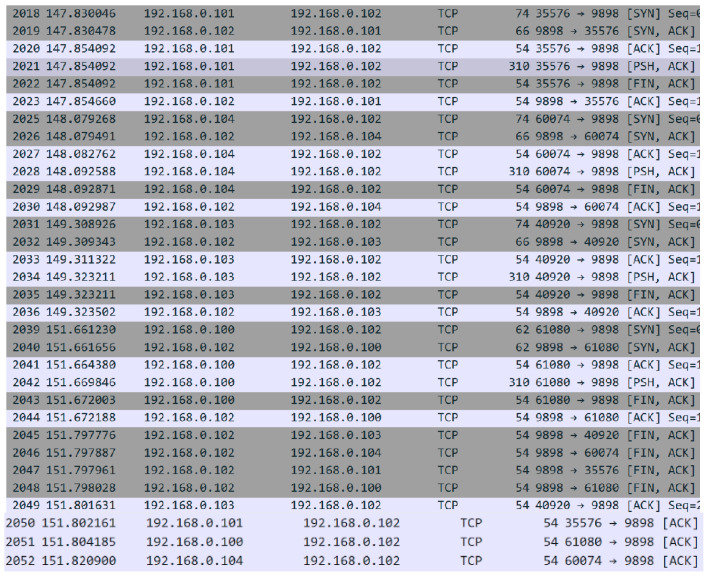
Final responses to the block creator (192.168.0.102) Wireshark network traffic.

Figure 22 displays the network traffic in Wireshark of the final response of the nodes to the block creator (192.168.0.102). This demonstrates the full network traffic for block approval’s final voting system.

### 5.3. Markov Model and Reputation Score

**Figure 23:**
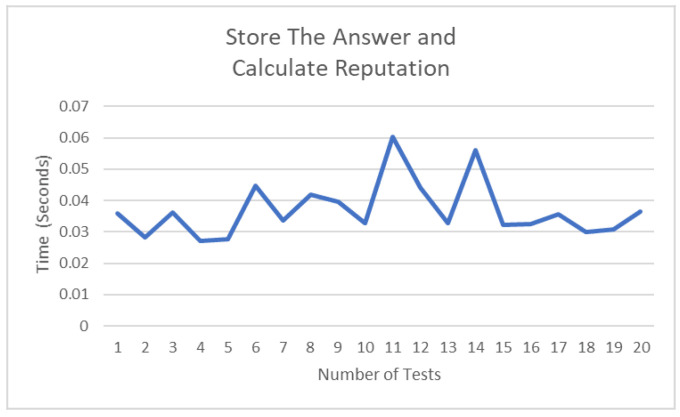
Time taken to calculate and store the reputation score of nodes.

Section 3.7 explains in detail the use of the Markov model and storage of reputation score that becomes very decisive in ensuring partition tolerance of the blockchain system. The example provided in that section clearly explains the procedures to follow and how the system calculate the reputation of each node. Figure 23 shows changes in time taken to calculate and store the new score, which can be caused by many factors, such as the current processes running on the machine or the complexity of the task.

### 5.4. RSA Encrypted Communication

**Figure 24:**
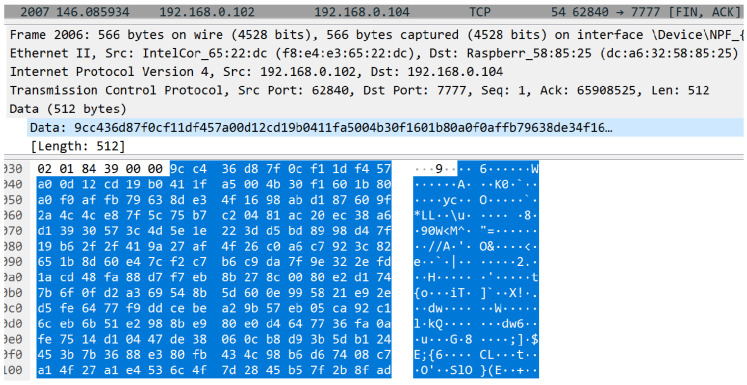
Wireshark snapshot of the encrypted data frame.

As elaborated in Section 3.6, secure communication of the nodes in the network with RSA 2048-bit key encryption is used to ensure the secure transfer of data within the network. Figure 24 demonstrates an encrypted data packet encrypted by 192.168.0.102 with its private key, sent to 192.168.0.104, where it would be decrypted using the sender’s pre-shared public key.

### 5.5. Merkle Tree

Implement error detection within the block content and the blockchain by using the Merkle tree proposed earlier in this paper in Section 3.11.1 to improve the prototype system. However, due to time constraints, it was not possible to implement this feature at this time.

### 5.6. Running on Unix-like Systems and Windows

The application is tested on multiple Unix-like operating systems such as Raspberry Pi, which is Debian-based, and Windows 10 operating systems. There are no compatibility issues as long as all the libraries are installed correctly and the directory of the files is identified.

### 5.7. Security Concerns

The implementation of the prototype takes into consideration common security concerns. The authentication of new nodes is dealt with through the genesis node; the use of asymmetric keys also ensures the authenticity of the private communication between nodes. In addition, once the new node decides to create new entries, verification of the digital signature of the data is required.

The secure communication of the nodes using RSA encryption with a 2048-bit key ensures that the data cannot be snooped or tampered with, essentially a method of threat prevention for likes of man-in-the-middle (MITM) attacks.

Minimum use of open ports is considered to lower the risk of vulnerabilities. As a result, only the ports that run at the start of the application are kept running, and all the other ports are closed immediately after use.

### 5.8. Network Traffic

Initially, during the consensus of block verification, each round of validation was sent separately, causing TCP congestion in the network. During the refinement of the prototype, the network traffic was considered; as a result, 10 verification rounds compressed into one list were implemented, demonstrated in Section 4.2.

### 5.9. Resource Usage

In terms of storage of the block in the Blockchain.txt, on average, each block takes around 229.24 bytes, depending on the block content.

**Figure 25:**
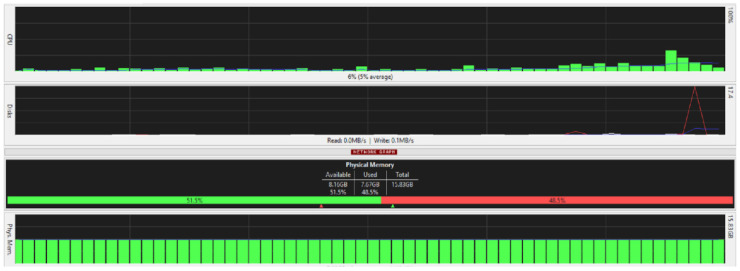
Running the monitoring tool before the application in process (block creator).

**Figure 26:**
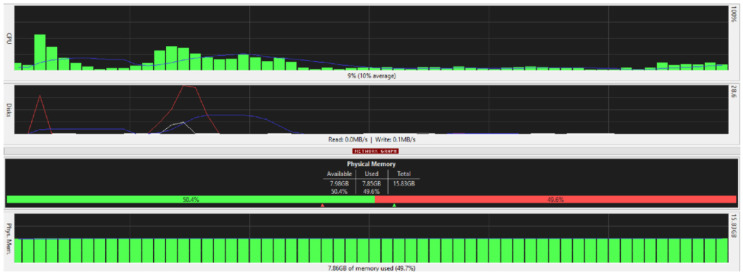
Running the monitoring tool after the application is in process (block creator).

**Figure 27:**
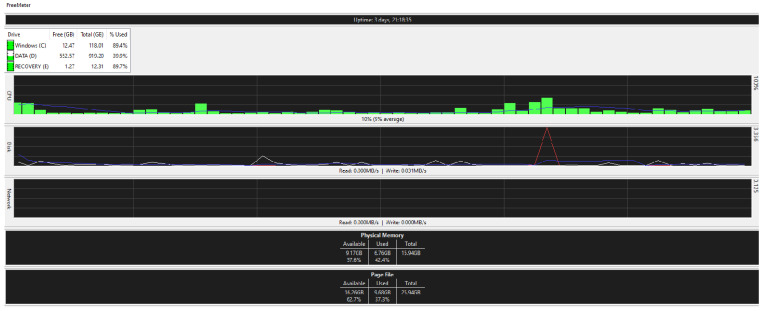
Running the monitoring tool before the application is in process (regular node).

**Figure 28:**
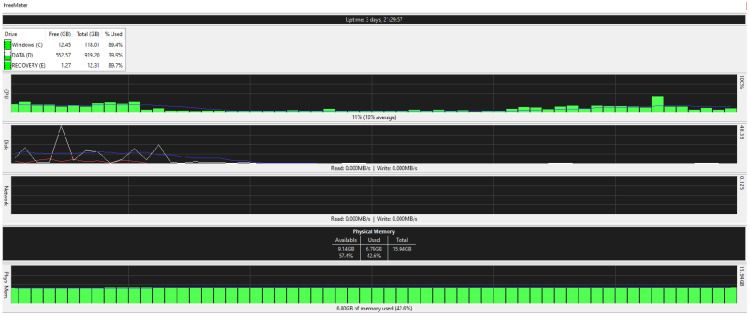
Running the monitoring tool after the application is in process (regular node) Windows machine.

Figure 25, Figure 26, Figure 27 and Figure 28 are taken from a network monitoring tool called the FreeMeter, capturing CPU usage, RAM usage, and disk use of the prototype for 60 s (segments of 10 s) with the application running at the beginning of the graph. This section demonstrates the resource usage while creating the block and participating in the consensus as a regular node.

#### 5.9.1. Block_Creator

From Figure 26, it can be seen that the physical memory or the RAM was the least affected; you can see a spike in disk use as the block creator initially reads encryption keys and local files to send the data, and once the responses are received, the system updates the stored data accordingly.

Figure 26 shows a spike in CPU usage surpassing 50% when the application starts running and the data are sent to the nodes in the network. Another spike occurs when the nodes respond.

#### 5.9.2. Regular_Node

Figure 27 and Figure 28 illustrate the resource usage of a regular node (192.168.0.100). Figure 27 shows a 60 s snapshot of the resource usage without the application running. Figure 28 is a snapshot of the resource usage in the same length of time with the prototype application running. Like the block creator, a spike in disk use and CPU usage is observable; in addition, slight changes to the RAM usage suggest that the application is not memory intensive.

The CPU usage is not specific to the application, and other processes running in the background impact the results of the test. Therefore, the taskset command is used on a Raspberry Pi node to find a more accurate result of the CPU usage.

**Figure 29:**
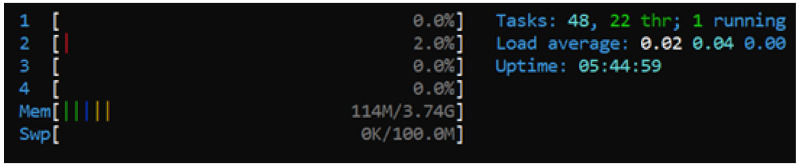
Using htop command to monitor RAM and CPU usage on a single core.

Using ps aux command, the process ID (PID) is identified, taskset -cp 3 (PID) command is used to assign the application process to the CPU core number 4. Finally, htop command can present the CPU core usage for the Raspberry Pi node. Figure 29 displays the data gathered from this tool, which shows that the consensus mechanism, although bearable, is CPU intensive for a short period of time.

**Figure 30:**
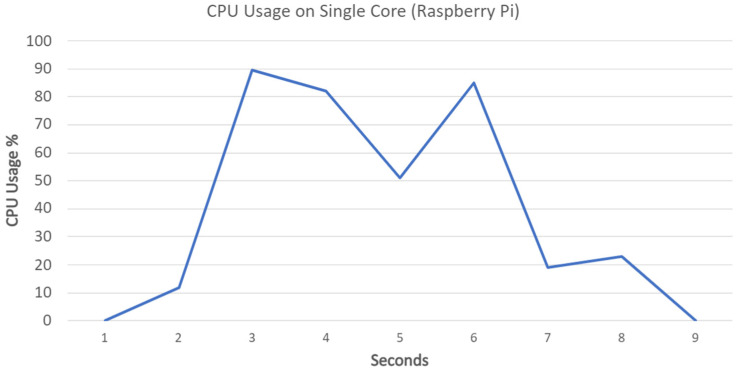
CPU usage on a single core (Raspberry Pi).

Figure 30 demonstrates the CPU usage on a single core, the initial rise in the usage is caused by data transmission, decryption followed by the verification (consensus) process. The CPU usage is handled smoothly by a single core of the Raspberry Pi in this case. No CPU failures occurred during the testing procedure.

### 5.10. Compatibility IDE and CLI

The system is tested to make it compatible with different execution scenarios. So, the application is expected to be compatible with any integrated development environment. To ensure its flexibility and adoptability during execution, the prototype is tested on various platforms, including: Thonny IDE, Spyder IDE, and command-line interface CLI.

### 5.11. Time Consumption to Create a Block

**Figure 31:**
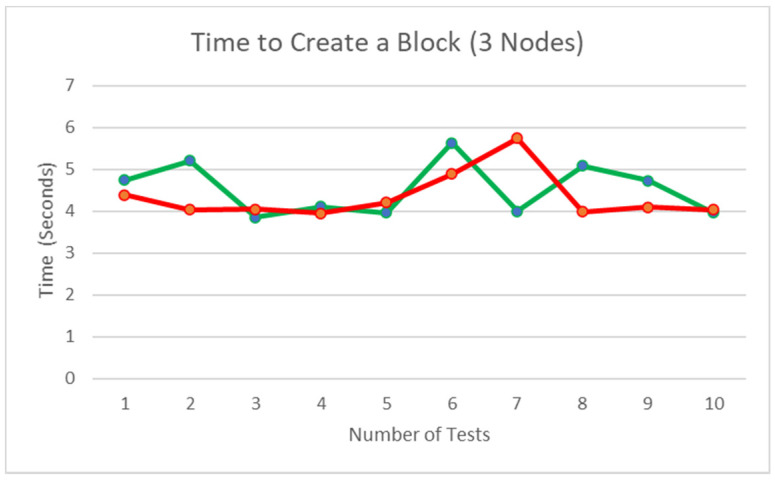
Time to create a block with 3 running nodes.

Figure 31 illustrates that the time is taken to create a block; from submitting the new block to when the block is validated and stored, averages about 4.43315513 s. The green line on the graph represents when all the nodes agree to the data to be submitted to the blockchain, and the red line on the chart represents the time taken when one node disagrees with the block content received, and their response is ‘False’, which results in the second part of the consensus to take place for the final decision. It is observed that the second part of the consensus has minimal impact on the overall block creation, with the green line average being 4.528968 s and the red line average being 4.337343025 s. Ten rounds of testing to obtain this result is justifiable as the results are almost similar, and we can obtain sufficient statistics from the number of tests carried out. The results range from 3.957380056 to 5.737800121 as the node waits for a response from all the nodes in the network. Considering the transfer of the block content, signature, and the verification process, the data obtained seem accurate.

**Figure 32:**
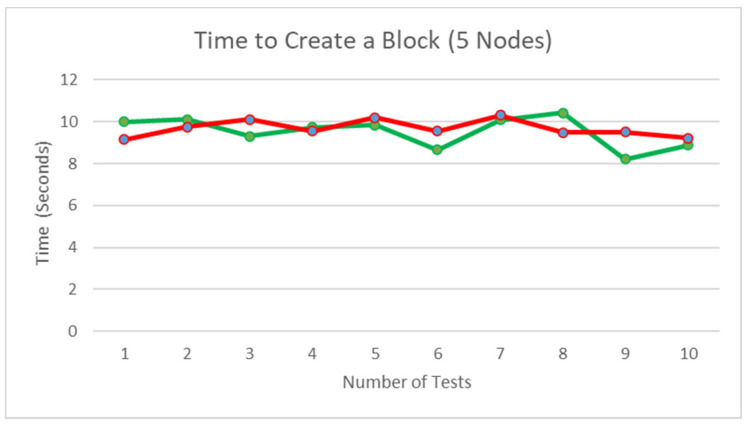
Time taken to create a block with 5 running nodes.

Figure 32 illustrates the time taken with five running nodes, an average of 9.518736 s taken for the nodes with no conflict in decision making and 9.680002 s taken for the nodes with conflict in decision resulting in the second part of the consensus to be activated. Similar to Figure 31, the results show that the conflict in decision has a minimal effect on the time taken for the block to be created; however, the number of nodes in the network has a positive correlation with the time taken to generate a block. Each node in the network participates in the consensus decision-making of the new block, so the positive correlation is caused by the response time by all the nodes during the verification process.

## 6. Discussion

The paper indicates the excellent potential of blockchain technology in distribution systems, particularly in the pharmaceutical industry, where the transparency, integrity, and validity of the data input are essential for monitoring agencies and fighting against fake medicine circulation to provide a sustainable healthcare system. The proposed solution initiates a novel contribution using decentralized technology to monitor the flow of products between entities within the pharmaceutical ecosystem. Overall, the prototype met all the aims, objectives, high priority, and most of the low-priority requirements. The blockchain system is created in a private network where the nodes go through various authentication to participate in the decision-making process, ensuring the integrity of the data input. The initial connection is through the genesis node, which then the new node receives all the required data to be able to participate in data entry to the system and the consensus protocol. The distributed ledger is accessible by all the participating nodes, and the communication between the nodes is secure using RSA encryption with a 2048-bit key. The implementation of the system is based on zero-knowledge; each node keeps records of the interactions they have with other nodes. Using the Markov model, each node is given a reputation score, which allows the system to make a better and more accurate final decision once the system participates in the decision-making process of accepting new entries. This also guarantees that only the nodes that are highly trustable participate in the consensus process during the block validation. The nodes whose trust values are low are rejected by the system from engaging in the mining process. This process provides scope for scalability for multiple reasons, including (a) All nodes will not participate during the consensus/mining process, and it fastens the validation process and provides scope to conduct more valid transactions per second (b) Invoking and involving only trusted nodes in the network improves the reliability of the validation process (c) Improves security and makes it resilient against 51% attack because the trusted nodes could be from anywhere and anyplace and it will be extremely hard to coordinate and cooperate in conducting the operation. One of the biggest issues pertaining to the blockchain is the high demand for energy consumption during block validation and block update process, and the proposed consensus mechanism attempt to address some of those issues and makes it more scalable. The application is acceptable to run on multiple operating systems such as Windows Unix-based OS and Debian-based operating system for Raspberry Pi, which demonstrates the adoptability of a naturally resource-hungry blockchain system in a low resource system link Raspberry Pi. Considerations were given to reducing network traffic in the consensus rounds. Finally, compatibility to run on different IDE and CLI were successfully tested. The consensus protocol of the proposed prototype with five nodes can take up to 9 s for creating a block, showing a substantial increase from the time taken with three nodes. This is a lot higher than the other blockchain networks such as Algorand and Ethereum (5 and 13 s, respectively) considering the transactions recorded in each block and the number of nodes; therefore, the system requires further adjustments for efficiency and speed of block-creation. However, it is important to note that the designed system is tested using a mix of standard PC configuration, a low powered and low configured Raspberry Pi, that is the reason for taking more time than expected in comparison to the other techniques and the most popular Bitcoin takes 10 min in average for a validation process.

## 7. Critical Evaluation

This section includes the limitations of the prototype system. Due to the lack of a high number of systems, the proposed model was tested only with a limited number of systems. All the systems are valid and are trustable, so an invalid node was intentionally inserted to mimic the existence of an un-trustable system in the blockchain network. In addition, other limitations include the fact that the nodes are identified by their IP address in this framework rather than using an anonymized public address. In a static DHCP network setting, the IP address can be changed or cloned; however, this work is for proof of feasibility study and designing an efficient consensus mechanism, so the focus is not on the authentication and identification process. In the future, an alternate method of authentication mechanism will be designed on a separate blockchain interlinked with the primary system.

In this framework, the genesis system’s public key is provided to all the participating nodes through a trusted channel (it is distributed to all the participating nodes). In fact, knowing or distributing the public key does not cause any harm since the corresponding private key is not known.

The current system waits for all the nodes in the system to complete their participation before resuming a process that consumes a long time; this is recognized by the time block creation tests in Section 5.11 when new nodes are added to the system. In the future, to make the system more efficient, the system could wait only for the responses of reputable nodes in the list that each entity stores for themselves in the reputation score list. In addition, the current proportionate of the reputable nodes are only 80% of the total nodes in the network. This could be problematic when the number of nodes is extremely high, and this is highly likely in pharmaceutical distribution, so for a solution as the number of nodes increases and surpasses a certain level, reputable node halving could be introduced, or only authorized certain nodes could be selected, such as the Binance network to improve the network validation process, or similar to the reward halving of the Bitcoin system. Because, in the pharmaceutical industry, all participants are trustable, accountable, and reliable, so using a smart contract, only a few trusted systems could be assigned, and a very high efficient ecosystem could be achieved.

The current system can only process consensus decisions for one block at a time, but to enhance the performance, multithreading execution in the multi-process could be designed to process multiple blocks simultaneously, and fast memory-accessing techniques could be adopted to improve the memory accessing time in the future. The nodes with inaccurate data must request the data content from the reputable nodes and update their blocks. Finally, the blockchain network requires the majority of the nodes in a network to be honest for the consensus to run accurately in most cases, but in the pharmaceutical industry, it may not be the case because all the participants of the supply chain have agreed to contracts, so fewer nodes can participate in the consensus process as highlighted earlier.

## 8. Conclusions and Future Work

The proposed solution provides a transparent flow of medicine between entities within the distribution system. Entities such as hospitals, pharmacies, and government bodies can trace their products back to the manufacturer to ensure the integrity and avoid counterfeit drugs and improve public health using a trust-based reputation decentralized system. The practitioners and even consumers can also verify medicines using API endpoints to such a system.

The system was designed to ensure consistency, availability, and partition tolerance from the CAP’s theorem; consistency of the data input throughout the network was provided using the ZKP proof. The system is always available if the network is not creating blocks simultaneously, causing override in the consensus rounds. The Markov model for scorekeeping of the nodes ensures a trust-based decision-making process. This designed system provides a scalable and trust-based consensus mining system to guarantee a traceable, transparent, and fault-tolerant pharmaceutical distribution system.

Future improvements require resolving the limitations mentioned in the previous section. The self-healing and problem detection using the Merkle tree will be designed in the future to make the system more resilient and robust.

## Figures and Tables

**Table 1:** Systems configuration.

**Device Configuration**					
**System**	**Desktop (192.168.0.100)**	**Raspberry Pi (192.168.0.101)** **Raspbian**	**Raspberry Pi (192.168.0.103)** **Raspbian**	**Raspberry Pi (192.168.0.104)** **Raspbian**	**Laptop (192.168.0.102)**
**OS**	Windows 10	Raspberry Pi OS	Raspberry Pi OS	Raspberry Pi OS	Windows 10
**CPU**	Core i5 5700	4× ARM Cortex-A53, 1.2 GHz	4× ARM Cortex-A53, 1.2 GHz	4× ARM Cortex-A53, 1.2 GHz	Core i7 10510U
**RAM**	16 GB	1 GB	1 GB	1 GB	16 GB
**Wireless Router Configuration**					
**Standards**	**Wi-Fi Speeds**	**Processor**	**Wi-Fi Transmission Power**	**Wi-Fi Encryption**	**Ethernet Ports**
IEEE 802.11n/b/g 2.4 GHz	2.4 GHz: 450 Mbps (802.11n)	Single-Core CPU	CE: <20 dBm(2.4 GHz) FCC: <30 dBm	WEP, WPA WPA2, WPA/WPA2-Enterprise (802.1x)	1× 10/100 Mbps WAN Port 4× 10/100 Mbps LAN Ports

## Data Availability

Not applicable.
